# A Comparison of Health Outcomes in Older versus Younger Adults following a Road Traffic Crash Injury: A Cohort Study

**DOI:** 10.1371/journal.pone.0122732

**Published:** 2015-04-01

**Authors:** Bamini Gopinath, Ian A. Harris, Michael Nicholas, Petrina Casey, Fiona Blyth, Christopher G. Maher, Ian D. Cameron

**Affiliations:** 1 John Walsh Centre for Rehabilitation Research, Sydney Medical School, University of Sydney, Sydney, Australia; 2 Ingham Institute for Applied Medical Research and South Western Sydney Clinical School, University of New South Wales, Kensington, Australia; 3 Pain Management Research Institute, Sydney Medical School, University of Sydney, Sydney, Australia; 4 School of Public Health, University of Sydney, Sydney, Australia; 5 George Institute for Global Health, Sydney Medical School, University of Sydney, Sydney, Australia; Griffith University, AUSTRALIA

## Abstract

**Background:**

Given the aging demographics of most developed countries, understanding the public health impact of mild/moderate road traffic crash injuries in older adults is important. We aimed to determine whether health outcomes (pain severity and quality of life measures) over 24 months differ significantly between older (65+) and younger adults (18–64).

**Methods:**

Prospective cohort study of 364, 284 and 252 participants with mild/moderate injury following a vehicle collision at baseline, 12 and 24 months, respectively. A telephone-administered questionnaire obtained information on socio-economic, pre- and post-injury psychological and heath characteristics.

**Results:**

At baseline, there were 55 (15.1%) and 309 (84.9%) participants aged ≥65 and 18–64 years, respectively. At 12- and 24-month follow-up, older compared to younger participants who had sustained a mild/moderate musculoskeletal injury had lower physical functioning (3.9-units lower Short Form-12 Physical Composite Score, multivariable-adjusted p = 0.03 at both examinations). After multivariable adjustment, older (n = 45) versus younger (n = 207) participants had lower self-perceived health status (8.1-units lower European Quality of Life-5 Dimensions Visual Acuity Scale scores at 24 months, p = 0.03), 24 months later.

**Conclusions:**

Older compared to younger participants who sustained a mild/moderate injury following a road-traffic crash demonstrated poorer physical functioning and general health at 24 months.

## Introduction

Globally, road traffic injuries are ranked twelfth in terms of contribution to disease burden as measured by Disability Adjusted Life Years [1]. Injured people with poor recovery generate the highest costs [2, 3]; therefore, it is important to understand the factors independently associated with poor outcomes following vehicle crash-related injuries. Published research indicates that the age-related decline in physical health increases the likelihood of poor outcomes among older drivers involved in a road traffic crash [4, 5]. Even a minor crash could have more serious implications for an older compared to a younger person suffering the same injuries [4, 5].

*Andersen et al*. [6] examined the differences in self-reported health as measured in the Short-Form-36 domains, among young (ages 18–64) compared to older (ages 65+) over 12 months post-injury. They showed that older versus younger adults with moderate/severe injuries had poorer general health. However, when age-associated conditions and pre-injury health were considered, age ceased to be a significant predictor of self-perceived general-health status. This study did not assess associations with milder injuries, which could also result in significant decreases in wellbeing [6]. Moreover, there is a lack of cohort studies that have made age-comparisons for pain severity and other health-status measures (e.g. European Quality of Life-5 Dimensions Visual Acuity Scale, EQ-5D VAS) following injury. Research in this area is also not without its methodological shortfalls such as non-standardized outcome measures and lack of adjustment for potential confounders such as pre-injury health [6].

Given that older drivers comprise the fastest growing segment of the Australian driving population [5], it is important to comprehensively characterize the long-term impact of a road traffic crash injury on the physical and psychological well-being of older adults. Therefore, this 24-month cohort study had the following key objectives: 1) Compare the socio-demographic, pre-injury health parameters, and injury-related parameters between older (65+) and younger (18–64) persons with mild/moderate musculoskeletal injuries following a vehicle-related crash; and 2) Determine whether significant differences in health outcomes between older and younger adults are present at 12 and 24 months, after adjusting for potential confounders.

## Methods

### Study population

Potential participants were identified from the New South Wales (NSW) Motor Accident Authority (MAA) Personal Injury Registry database. The MAA is the government regulator of companies providing third party motor vehicle accident insurance in NSW. This database consists of people who made claims on the Compulsory Third Party scheme. Claimants aged ≥18 years who had sustained injuries in a motor vehicle crash in NSW between March and December 2010 were identified and invited to participate in the study. Participants were excluded if they: a) sustained severe injuries (severe traumatic brain injury or spinal cord injury; b) had an injury requiring hospitalization for more than 7 days; c) had a New Injury Severity Score (NISS) >8; c) were unable to complete questionnaires by telephone in English; and/or e) if contact could not be initiated within 60 days of the crash date.

**Fig 1:**
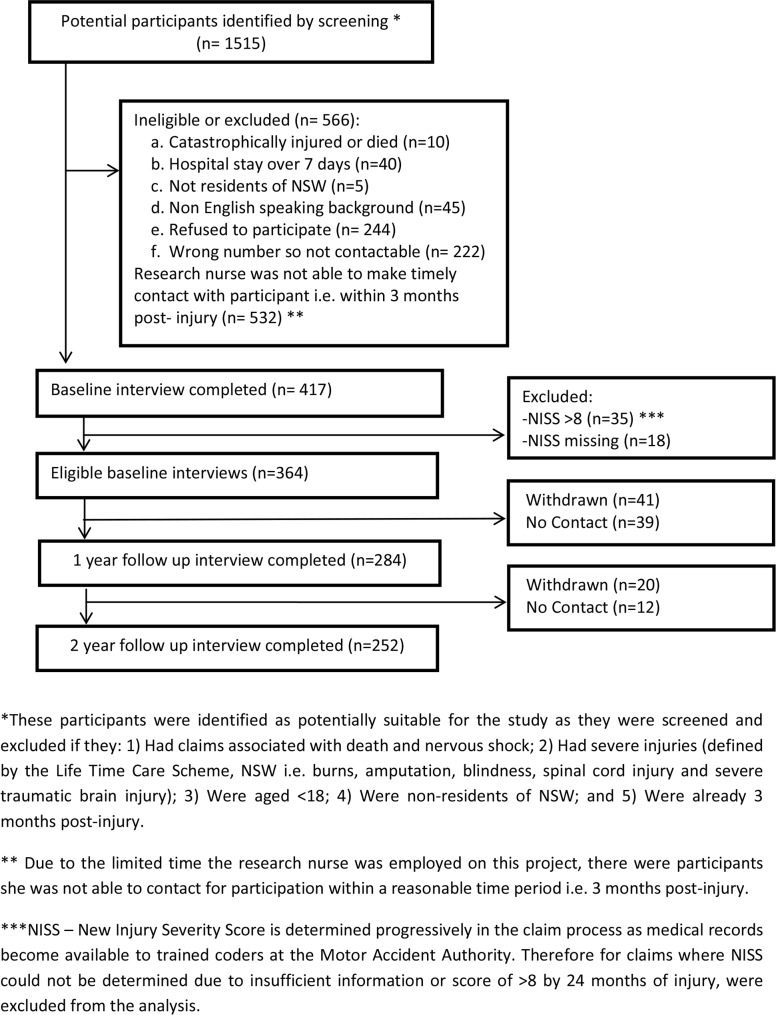
Flowchart of study participation.

A total of 1,515 insurance claims that were lodged between March 2010 and December 2010 were deemed to be potential participants (Fig 1), and these individuals were sent a letter of invitation by the MAA together with the Participant Information Sheet. An opportunity to opt out of the study within 2 weeks was provided, following which, verbal consent was sought. As the survey was conducted over the phone, obtaining verbal consent was deemed to be appropriate. Completion of the survey was documented as giving consent to participate and this was recorded by the research nurse who administered the survey. This study (including use of verbal consent) was approved by the University of Sydney health research ethics committee. This study was conducted according to the principles expressed in the Declaration of Helsinki.

### Assessment of social and health characteristics

Participants in the study were interviewed by telephone on average 56 (range 25–92) days following the date of the accident. While the initial contact with the injured person was within 60 days of the crash, a number of follow-up calls were needed for the first available date for the actual interview, which extended this to a maximum of 92 days post-injury. The interview schedule was structured and used a closed response format. All interviews were administered by one trained and experienced research nurse. The Abbreviated Injury Scale coding system was used to classify the participants into: mild (1–3) and moderate (4–8) musculoskeletal injury groups based on the NISS [7]. Around 17 trained and experienced coders were used to code the reported injuries.

Chronic illness was determined by asking participants if they had been diagnosed with any of the following: asthma, cancer, heart/circulatory condition, diabetes, mental and behavioral problems, and/ or other in the last three months. Chronic pain was characterized by participants reporting that they had been diagnosed with the following for more than 3 months: arthritis, neck and back problems/disorder, or pain.

Participants were asked to describe their general health status prior to the accident, using a 5-point Likert scale. Body mass index (BMI) was calculated from self-reported height and weight, and classified according to WHO guidelines: <20 kg/m^2^ (underweight), 20–24.9 kg/m^2^ (normal), 25–29.9 kg/m^2^ (overweight), ≥30 kg/m^2^ (obese). Participants were also asked how many hours that they spent in hospital after the crash.

The Pain-Related Self-Statements Scale-Catastrophizing Subscale (PRSS-Catastrophizing) measures the frequency of a patient's catastrophic cognitions that may impede the individual's ability to cope with severe pain [8]. Patients are asked to rate the frequency with which they experience catastrophic thoughts during an episode of pain, and the overall score is calculated with a range of 0 (‘almost never’) to 5 (‘almost always’) [8, 9].

The short-form Orebro Musculoskeletal Pain Screening Questionnaire (OMPSQ) allows for the early identification of persons likely to develop persistent disability from musculoskeletal pain affecting return to work [10]. A score above 50 (out of 100) identifies individuals at high risk of developing poor return to work outcomes, and scoring below 50 is classified as low risk [10].

### Assessment of health outcomes

European Quality of Life-5 Dimensions (EQ-5D) scale was used to measure health-related quality of life [11]. The first part of the EQ-5D has five dimensions: mobility, self-care, usual activities, pain/discomfort and anxiety/depression. Each dimension is divided into: no problem, some problems and major problems. The second part is a 20-cm visual analogue scale (EQ-5D VAS), which was modified slightly from the original version with a repetition of the question: ‘To help you say how good or bad your health state is, I have a scale in front of me (rather like a thermometer), on which the best health state you can imagine is marked 100 and the worst health state you can imagine is marked 0’ [11, 12]. The Short Form-12 (SF-12) was another measure of quality of life [13]. The SF-12 has 12 questions selected from the SF-36 [14]. Two summary scores, the physical and mental component summaries, are derived from 8 domain scores, the domain scores and component scores are standardized to a normal Australian population mean of 50 and standard deviation of 10. An average overall pain severity was assessed using a 0 (‘no pain’) to 10 (‘worst pain imaginable’) numeric rating scale (NRS).

### Statistical analysis

Baseline characteristics of older compared to younger adults were summarized using descriptive statistics and differences in study characteristics were compared using the chi-square test or analysis of variance where appropriate. Relationships between age-group in the sub-acute phase and various study outcomes were assessed separately at 12 and 24 months using linear regression. Univariate analysis was used to identify significant risk factors (i.e. two-tailed *p* <0.05) for each study outcome at 12 and 24 months. Potential covariates that were considered included: sex, marital status, education level, pre-injury paid work status, BMI, pre-injury health variables (health status, chronic illnesses and chronic pain), hospital admission, NISS score, whiplash (due to the car crash), fracture (due to the car crash), OMPSQ score, and PRSS score. Multivariable analyses selected factors significantly associated with each specific study outcome (SF-12 PCS and MCS, EQ-5D VAS and pain severity rating), using a backward elimination procedure for excluding variables without associations with the study outcome of interest (p >0.05). Therefore, only adjustment variables significant at the 5% level were retained in the final model, although, sex was included in all models irrespective of its level of significance. Also, the final models were determined after evaluating the likely causal pathway and goodness of fit. At 12 months, subjects included in the analysis were participants who responded to both interviews at baseline and the follow-up. This was also applicable at 24-month assessment. Due to the loss to follow-up, the number of participants at both assessments was different. Significance level was p <0.05. Statistical analyses were done using SPSS v 21 (IBM SPSS Incorporated, Chicago, IL, USA).

## Results

Of the 1515 potential participants, 1098 were not eligible or refused to participate (Fig 1). Of the remaining 417 who participated in the baseline interview (i.e. sub-acute phase), 53 were excluded as they had missing NISS or an NISS >8 (severe injury). This left 364 participants that could be included in analyses. Twelve- and 24-month follow-up assessments were completed on 284 (78% of eligible participants in the sub-acute phase) and 252 (69% follow-up rate) of 364 enrolled and eligible participants, respectively (Fig 1). Of the 364 participants who were surveyed at baseline, 284 participants provided follow-up data at 12 months. Study characteristics of follow-up responders versus non-responders (i.e. those who were lost to follow-up at 12 months) were compared (S1 Table). Responders compared to non-responders were likely to be older.

At baseline, there were 55 (15.1%) and 309 (84.9%) participants aged ≥65 and <65, respectively. Participants aged ≥65 versus <65 were less likely to: have tertiary qualifications, pre-injury paid work, excellent/very good pre-injury health status, whiplash (sustained in the car crash), OMPSQ score >50 and PRSS score ≥3 (Table 1). However, older versus younger participants were more likely to be admitted to hospital (≥1 night), and have pre-injury chronic illness.

**Table 1:** Socio-demographic, psychological, health and injury-related characteristics of participants in the sub-acute phase, stratified by age-group (n = 64).

**Parameters**	**Older (≥65 yrs) n = 55**	**Younger (18–64 yrs) n = 309**	**P-value**
Sex			0.28
Male (n = 135)	24 (44%)	111 (36%)	
Female (n = 229)	31 (56%)	198 (64%)	
Education^*^			0.04
Tertiary qualified (n = 100)	9 (16%)	91 (30%)	
Not tertiary qualified (n = 263)	46 (84%)	217 (71%)	
Paid work status			<0.001
Yes (n = 227)	5 (9%)	222 (72%)	
No (n = 137)	50 (91%)	87 (28%)	
Body mass index			0.98
Underweight (n = 28)	4 (7%)	24 (8%)	
Normal (n = 128)	19 (35%)	109 (35%)	
Overweight/ obese (n = 208)	32 (58%)	176 (57%)	
Smoking			0.05
Yes (n = 51)	3 (6%)	48 (16%)	
No (n = 311)	52 (95%)	259 (84%)	
Pre-injury health status			0.005
Excellent/ very good (n = 274)	32 (58%)	242 (78%)	
Good (n = 67)	18 (33%)	49 (16%)	
Fair/ poor (n = 23)	5 (9%)	18 (6%)	
Pre-injury chronic illness			<0.001
No (n = 218)	16 (29%)	202 (65%)	
Yes (n = 146)	39 (71%)	107 (35%)	
Pre-injury chronic pain			0.41
No (n = 311)	45 (82%)	266 (86%)	
Yes (n = 53)	10 (18%)	43 (14%)	
New injury severity scale			0.24
Mild (n = 310)	44 (80%)	266 (86%)	
Moderate/ severe (n = 54)	11 (20%)	43 (14%)	
Admitted to hospital (≥1 night)			0.04
No (n = 295)	39 (71%)	256 (83%)	
Yes (n = 69)	16 (29%)	53 (17%)	
Whiplash (due to the car crash)			<0.001
No (n = 139)	35 (64%)	104 (34%)	
Yes (n = 224)	20 (36%)	204 (66%)	
Fracture (due to the car crash)			0.77
No (n = 333)	51 (93%)	282 (92%)	
Yes (n = 30)	4 (7%)	26 (8%)	
OMPSQ score			0.01
≤50 (n = 207)	40 (73%)	167 (54%)	
>50 (n = 157)	15 (27%)	142 (46%)	
PRSS score			0.03
<3 (n = 306)	50 (94%)	256 (83%)	
≥3 (n = 56)	3 (6%)	53 (17%)	

Data are presented as n (%); OMPSQ—Orebro Musculoskeletal Pain Screening Questionnaire; PRSS- Pain-Related Self-Statements Scale-Catastrophizing.

At 12- and 24-month follow-up (Tables 2 and 3), older compared to younger participants who had sustained a mild/moderate musculoskeletal injury had lower physical functioning (3.9-units lower SF-12 PCS score, multivariable-adjusted p = 0.03 at both examinations). There was also a marginally significant difference (multivariable-adjusted p = 0.06) in EQ-5D VAS scores between older and younger adults (Table 2). After multivariable adjustment, older (n = 45) versus younger (n = 207) participants had lower self-perceived health status (8.1-units lower EQ-5D VAS scores at 24 months, p = 0.03), 24 months later (Table 3).

**Table 2:** Quality of life scores and severity of pain among older (≥65 years) and younger (18–64 years) participants 12 months after a mild/ moderate musculoskeletal injury (n = 284).

	**Estimated marginal means (95% CI)**		
**Health outcome**	**Older (n = 48)**	**Younger (n = 236)**	**P-value**
SF-12 physical component score ^a^	37.0 (33.3–40.6)	40.9 (38.8–42.9)	0.03
SF-12 mental component score ^b^	49.7 (46.5–52.8)	49.4 (48.1–50.7)	0.89
EQ-5D Visual Analogue Score ^c^	57.5 (52.1–63.0)	62.9 (59.6–66.1)	0.06
Pain numeric rating scale score (NRS)^d^	5.45 (4.61–6.29)	5.03 (4.68–5.39)	0.37

^a^ Adjusted for sex, pre-injury general health, pre-existing chronic illness, education, whiplash (due to the car crash) and baseline Orebro Musculoskeletal Pain Screening Questionnaire (OMPSQ) score.

^b^ Adjusted for sex, smoking, pre-existing chronic illness, whiplash, baseline Pain-Related Self-Statements Scale-Catastrophizing (PRSS) score and baseline OMPSQ score.

^c^ Adjusted for sex, pre-injury general health, whiplash (due to the car crash), baseline PRSS score and baseline OMPSQ score.

^d^ Adjusted for sex, education, fracture (due to the car crash), baseline PRSS score and baseline OMPSQ score.

**Table 3:** Quality of life scores and severity of pain among older (≥65 years) and younger (18–64 years) participants 24 months after a mild/ moderate musculoskeletal injury (n = 252).

	**Estimated marginal means (95% CI)**		
**Health outcome**	**Older (n = 45)**	**Younger (n = 207)**	**P-value**
SF-12 physical component score ^a^	37.7 (33.9–41.4)	41.6 (39.5–43.7)	0.03
SF-12 mental component score ^b^	44.9 (41.6–48.2)	47.8 (45.6–50.0)	0.08
EQ-5D Visual Analogue Score ^c^	56.8 (49.6–64.0)	64.9 (61.3–68.6)	0.03
Pain numeric rating scale score (NRS)^d^	5.25 (4.29–6.21)	4.59 (4.15–5.04)	0.23

^a^ Adjusted for sex, pre-injury general health, pre-existing chronic illness, baseline Pain-Related Self-Statements Scale-Catastrophizing (PRSS) score and baseline Orebro Musculoskeletal Pain Screening Questionnaire (OMPSQ) score.

^b^ Adjusted for sex, BMI, pre-injury general health, baseline PRSS score and baseline OMPSQ score.

^c^ Adjusted for sex, paid work, pre-injury general health, whiplash (due to the car crash), baseline PRSS score and baseline OMPSQ score.

^d^ Adjusted for sex, baseline PRSS score and baseline OMPSQ score.

## Discussion

This cohort study adds to the literature by establishing how older and younger adults with mild/moderate musculoskeletal injury following a motor-vehicle accident differ in terms of social and health parameters. Moreover, older compared to younger participants had slightly poorer self-perceived physical functioning and general health 12 and 24 months after the vehicle-related injury, respectively. The observed magnitude of difference in SF-12 PCS and EQ-5D VAS scores between older and younger participants was ~3–8 units after 24 months. This is within the range of 3–10 points which was previously defined as a meaningful difference in quality of life scores in a clinical setting [15]. These observed associations were independent of demographic and pre-injury health indicators.

Claimants aged ≥65 versus <65 demonstrated differences in a range of health and social parameters. Given their older age, it was not surprising that fewer than 10% were in paid work prior to the injury and that over three quarters did not have tertiary qualifications. Also, with age the number of chronic conditions increases [16] as reflected in around 71% of older adults self-reporting pre-injury chronic diseases. Pre-injury chronic illnesses in older adults could also have predisposed them to poorer pre-injury health. We also need to highlight that the prevalence of whiplash sustained in a road traffic crash in older compared to younger adults was exactly half (31% versus 62%). Other studies [17, 18] have shown that younger age is associated with a higher prevalence of crash-related injuries for neck pain in comparison with older age. *Freeman et al*. [17] posited that it was due to a lack of competition from other causes of pain, particularly insidious onset of pain, which was observed to be directly related to age (i.e. pain due to degenerative disc and joint disease).

It was interesting to note that fewer older versus younger adults scored ≥3 on the pain catastrophizing scale (5% versus 17%), indicating that this age-group was less troubled by their pain in the sub-acute phase compared to their younger counterparts. For pain catastrophizing, data on the impacts of age is limited [19], but there are some Australian data that indicate slightly higher rates of pain catastrophizing in patients with chronic pain aged <60 years of age versus those aged >60 [9]. Depression ratings in chronic pain samples have also been found to be significantly lower in the >60 age-group (versus <60) [20]. These findings suggest that older people in pain tend to be more stoic than their younger counterparts. Consistent with this, *Ruscheweyh et al*. [19, 21] showed that in younger adults, catastrophizing is associated with emotional response to pain while in older subjects, it is preferentially associated with the actual pain intensity. That is, older adults have more access to emotion regulation strategies than younger adults and consequently experience fewer negative emotions and lesser arousal in response to negative emotions [21, 22]. This could partly explain our observed differences between the age groups. However, further injury cohort studies are required to both confirm our observation and clarify underlying mechanism(s). Consistent with the pain catastrophizing findings, a lower proportion of older versus younger subjects scored ≥50 on the OMPSQ, indicating that the older age-group had lower psychosocial risk factors for developing persistent disability from musculoskeletal pain.

In relation to differences in health status between older and younger adults, comparing our results to those of studies published to date proves difficult. Most cohort studies have examined the effects of age on health status following a severe/traumatic vehicle-related injury. A significant but modest difference in SF-12 PCS scores or poorer physical functioning was observed between older and younger adults at 12 and 24 months. However, this association may not be clinically meaningful and could simply reflect age-related differences in PCS scores irrespective of injury; particularly, as it has been previously reported that norms for the SF-12 PCS are statistically significantly lower in older age groups [23]. This could also explain why data from a 1-year study of severely or moderately injured participants showed non-significant differences in SF-36 scores between older and younger adults [6]. On the other hand, it could be because our study included mild injuries, which in the elderly or the frail in particular, could lead to significant decreases in functioning if the injuries compromise mobility (e.g. ankle fractures) [6].

Participants aged ≥65 versus <65 years had a lower EQ-5D VAS score at 24 months. Poorer self-perceived health observed in older compared to younger participants cannot be fully explained with our data, because there are a number of biological (e.g. markers of frailty syndrome) and environmental factors (e.g. social support network) associated with age which could underlie this observation. It is also not clear why significant differences in EQ-5D VAS scores between younger and older participants emerged only at 24 months post-injury; these findings could be due to chance and should be interpreted with caution. Nevertheless, we hypothesize that physical capabilities (evidenced by low PCS scores) could be predominantly affected by age in the short-term after the injury, and that in the longer-term, psychosocial impacts captured by tools such as the EQ-5D VAS could be more strongly influenced by age.

Interpretation of study findings should be tempered with the caveat that we collected information on chronological age only. Biological age or underlying frailty could be a better indicator of a person’s ability to recover from an injury [6], hence, there is the potential for residual confounding from unmeasured age-related parameters. Other study limitations include the use of compensation system data. Compensable persons might not represent the broader injury population; hence, we cannot disregard selection bias. Further, recall bias may have been more pronounced in our study due to the 3-month delay (or sometimes more before participants were interviewed, even though initial contact was made by 3 months) between the injury event and measurement of recalled pre-injury characteristics. Additionally, participants compared to non-participants (those not followed up at either 12 and/ or 24 months) differed in age, hence, we cannot disregard the possibility of selection bias influencing observed associations. Finally, because we have examined several associations, the possibility of chance findings cannot be excluded. Despite these shortcomings, our study has several strengths including its longitudinal design, and rich data collected on a wide range of potential confounders.

## Conclusions

In summary, the knowledge gained from this study of differences in health determinants and health outcomes across the age spectrum adds to existing literature regarding factors impacting recovery from mild or moderate musculoskeletal injury sustained in a road traffic crash. Our study showed that older compared to younger adults who sustained a mild/ moderate injury following a road-traffic crash demonstrated poorer physical functioning and general health at 24 months. If our findings are confirmed by other studies, it will highlight the need for interventions targeting enhanced recognition and management of poorer physical functioning and overall health status in older compared to younger adults following a road traffic crash.

## Supporting Information

S1 Dataset Study population dataset. (SAV)

S1 Table Study characteristics of participants who were followed up compared to those not followed up at 12 months. (DOC)

## References

[1] Murray CJ, Vos T, Lozano R, Naghavi M, Flaxman AD, Michaud C, et al Disability-adjusted life years (DALYs) for 291 diseases and injuries in 21 regions, 1990–2010: a systematic analysis for the Global Burden of Disease Study 2010. Lancet. 2012;380: 2197–2223. 10.1016/S0140-6736(12)61689-4 23245608

[2] Littleton SM, Cameron ID, Poustie SJ, Hughes DC, Robinson BJ, Neeman T, et al The association of compensation on longer term health status for people with musculoskeletal injuries following road traffic crashes: emergency department inception cohort study. Injury. 2011;42: 927–933. 22081822 10.1016/j.injury.2010.02.011

[3] Connelly LB, Supangan R. The economic costs of road traffic crashes: Australia, states and territories. Accident; analysis and prevention. 2006;38: 1087–1093. 16797462 10.1016/j.aap.2006.04.015

[4] Li G, Braver ER, Chen LH. Fragility versus excessive crash involvement as determinants of high death rates per vehicle-mile of travel among older drivers. Accident; analysis and prevention. 2003;35: 227–235. 12504143 10.1016/s0001-4575(01)00107-5

[5] Meuleners LB, Harding A, Lee AH, Legge M. Fragility and crash over-representation among older drivers in Western Australia. Accident; analysis and prevention. 2009;38: 1006–1010. 10.1016/j.aap.2006.04.005 16713982

[6] Andersen D, Ryb G, Dischinger P, Kufera J, Read K. Self-reported health indicators in the year following a motor vehicle crash: a comparison of younger versus older subjects. Annals of advances in automotive medicine / Annual Scientific Conference Association for the Advancement of Automotive Medicine Association for the Advancement of Automotive Medicine Scientific Conference. 2010;54: 359–367. PMC3242549 21050618

[7] Stevenson M, Segui-Gomez M, Lescohier I, Di Scala C, McDonald-Smith G. An overview of the injury severity score and the new injury severity score. Injury prevention: journal of the International Society for Child and Adolescent Injury Prevention. 2001;7: 10–13. 11289527 10.1136/ip.7.1.10 PMC1730702

[8] Flor H, Behle DJ, Birbaumer N. Assessment of pain-related cognitions in chronic pain patients. Behaviour research and therapy. 1993;31: 63–73. 8417730 10.1016/0005-7967(93)90044-u

[9] Nicholas MK, Asghari A, Blyth FM. What do the numbers mean? Normative data in chronic pain measures. Pain. 2008;134: 158–173. 17532138 10.1016/j.pain.2007.04.007

[10] Linton SJ, Nicholas M, MacDonald S. Development of a short form of the Orebro Musculoskeletal Pain Screening Questionnaire. Spine. 2011;36: 1891–1895. 10.1097/BRS.0b013e3181f8f775 21192286

[11] EuroQol—a new facility for the measurement of health-related quality of life. Health policy (Amsterdam, Netherlands). 1990;16: 199–208. 10109801 10.1016/0168-8510(90)90421-9

[12] McPhail S, Lane P, Russell T, Brauer SG, Urry S, Jasiewicz J, et al Telephone reliability of the Frenchay Activity Index and EQ-5D amongst older adults. Health and quality of life outcomes. 2009;7: 48 10.1186/1477-7525-7-48 19476656 PMC2695435

[13] Ware JE SK, Kolinski M, Gandeck B. SF-36 Health survey manual and interpretation Guide Boston: Boston: The Health Institute, New England Medical Centre 1993

[14] Brazier J, Roberts J, Deverill M. The estimation of a preference-based measure of health from the SF-36. Journal of health economics. 2002;21: 271–292. 11939242 10.1016/s0167-6296(01)00130-8

[15] Samsa G, Edelman D, Rothman ML, Williams GR, Lipscomb J, Matchar D. Determining clinically important differences in health status measures: a general approach with illustration to the Health Utilities Index Mark II. PharmacoEconomics. 1999;15: 141–155. 10351188 10.2165/00019053-199915020-00003

[16] Hoffman C, Rice D, Sung HY. Persons with chronic conditions. Their prevalence and costs. JAMA: the journal of the American Medical Association. 1996;276: 1473–1479. 8903258

[17] Freeman MD, Croft AC, Rossignol AM, Centeno CJ, Elkins WL. Chronic neck pain and whiplash: a case-control study of the relationship between acute whiplash injuries and chronic neck pain. Pain research & management: the journal of the Canadian Pain Society = journal de la societe canadienne pour le traitement de la douleur. 2006;11: 79–83. 10.1155/2006/304673 PMC2585479 16770448

[18] Hijioka A, Narusawa K, Nakamura T. Risk factors for long-term treatment of whiplash injury in Japan: analysis of 400 cases. Archives of orthopaedic and trauma surgery. 2001;121: 490–493. 11599748 10.1007/s004020100284

[19] Leung L. Pain catastrophizing: an updated review. Indian journal of psychological medicine. 2012;34: 204–217. 10.4103/0253-7176.106012 23441031 PMC3573569

[20] Wood BM, Nicholas MK, Blyth F, Asghari A, Gibson S. The utility of the short version of the Depression Anxiety Stress Scales (DASS-21) in elderly patients with persistent pain: does age make a difference? Pain medicine (Malden, Mass). 2010;11: 1780–1790. 10.1111/j.1526-4637.2010.01005.x 21134119

[21] Ruscheweyh R, Nees F, Marziniak M, Evers S, Flor H, Knecht S. Pain catastrophizing and pain-related emotions: influence of age and type of pain. The Clinical journal of pain. 2011;27: 578–586. 10.1097/AJP.0b013e31820fde1b 21368662

[22] Carstensen LL, Isaacowitz DM, Charles ST. Taking time seriously. A theory of socioemotional selectivity. The American psychologist. 1999;54: 165–181. 10199217 10.1037//0003-066x.54.3.165

[23] Avery J DGE, Taylor A. Quality of life in South Australia as measured by the SF-12 Health Status Questionnaire: population norms for 2003: trends from 1997–2003 South Australia: Dept. of Human Services. Population Research and Outcome Studies Unit 2004

