# Supplementary material for: A Comparison of Health Outcomes in Older versus Younger Adults following a Road Traffic Crash Injury: A Cohort Study
Source: PLoS One. 2015 Apr 1;10(4):e0122732. doi: 10.1371/journal.pone.0122732 (PMC4382341; doi:10.1371/journal.pone.0122732)
Supplement: S1 Table — (DOC) [file pone.0122732.s002.doc]

**Supplementary Table 1.** Study characteristics of participants who were followed up compared to those not followed up at 12 months

| Characteristics | Participants  (n=284) | Non-participants ^a^  (n=80) | P-value |
| --- | --- | --- | --- |
| Age (years), mean (SD) | 46.4 (17.1) | 41.6 (14.6) | 0.02 |
| Male Sex, n (%) | 101 (35.6) | 34 (42.5) | 0.29 |
| Tertiary qualified ^b^, n (%) | 80 (28.3) | 20 (25.0) | 0.58 |
| Overweight/obese (≥25 kg/m^2^), n (%) | 157 (55.3) | 51 (63.8) | 0.20 |
| Excellent/very good pre-injury health, n (%) | 210 (73.9) | 64 (80.0) | 0.31 |
| No pre-injury chronic illness, n (%) | 166 (58.5) | 52 (65) | 0.30 |
| No pre-injury chronic pain, n (%) | 244 (85.9) | 67 (83.8) | 0.72 |
| Admitted to hospital (≥1 night), n (%) | 55 (19.4) | 14 (17.5) | 0.75 |
| NISS, mean (SD) | 2.4 (1.5) | 2.3 (1.7) | 0.48 |
| Whiplash, n (%) | 175 (61.8) | 49 (61.3) | 1.00 |
| Fracture, n (%) | 26 (9.2) | 4 (5.0) | 0.26 |
| Pain numeric rating scale, mean (SD) | 5.2 (2.5) | 5.5 (2.5) | 0.43 |
| OMPSQ score, mean (SD) | 43.0 (25.2) | 44.8 (23.8) | 0.57 |
| SF-12 PCS, mean (SD) | 36.9 (11.5) | 36.1 (11.6) | 0.60 |
| SF-12 MCS, mean (SD) | 46.7 (11.4) | 46.4 (10.9) | 0.85 |
| EQ-5D VAS, mean (SD) | 66.0 (21.7) | 65.0 (23.7) | 0.71 |

NISS – New Injury Severity Score; OMPSQ - Orebro Musculoskeletal Pain Screening Questionnaire; PCS – Physical Component Score; MCS – Mental Component Score; VAS – Visual Analogue Scale.

^a^ Non-participants only participated at baseline and did not further participate at 12 or 24 month follow-up surveys.

^b^ Bachelor degree or more is defined as tertiary qualified
